# Unique diversity of radioactive particles found in the Yenisei River floodplain

**DOI:** 10.1038/s41598-017-11557-7

**Published:** 2017-09-11

**Authors:** Alexander Bolsunovsky, Mikhail Melgunov, Alexey Chuguevskii, Ole Christian Lind, Brit Salbu

**Affiliations:** 1 Institute of Biophysics SB RAS, FRC Krasnoyarsk Science Center SB RAS, Krasnoyarsk, 660036 Russia; 2 Institute of Geology and Mineralogy SB, Russian Academy of Sciences, Novosibirsk, 630090 Russia; 3 Norwegian University of Life Sciences, Centre for Environmental Radioactivity, Ås, Norway

## Abstract

The long-term operation of three reactors and the radiochemical plant of the Mining-and-Chemical Combine (MCC), Russia’s largest producer of weapons-grade plutonium, has resulted in radioactive contamination of the Yenisei River floodplain. From 1995 to 2016, we found more than 200 radioactive particles (RP) in the Yenisei floodplain, downstream of the MCC. Analytical characterization showed that most of the RP were fuel particles, which were carried into the river after incidents at the MCC reactors. Having compared the ^137^Cs/^134^Cs ratios in the particles, we determined three time intervals when the RP were formed. The plutonium isotope ratios (^238^Pu/^239,240^Pu) vary substantially between the particles and indicate several different source terms. In addition to fuel RP, we found particles that only contained activation products (^60^Co or europium isotopes). SEM and γ-spectrometry showed that the cobalt particles could have originated from the corrosion of the reactor coolant system and the europium particles – from the damaged compensating rods. No europium particles have been found anywhere else in the world. The presence of RP from different sources (fuel, cobalt, and europium particles) in the Yenisei River floodplain makes this region a unique site for studying environmental effects of the particles. These RP represent point sources of radioecological significance.

## Introduction

**Figure 1:**
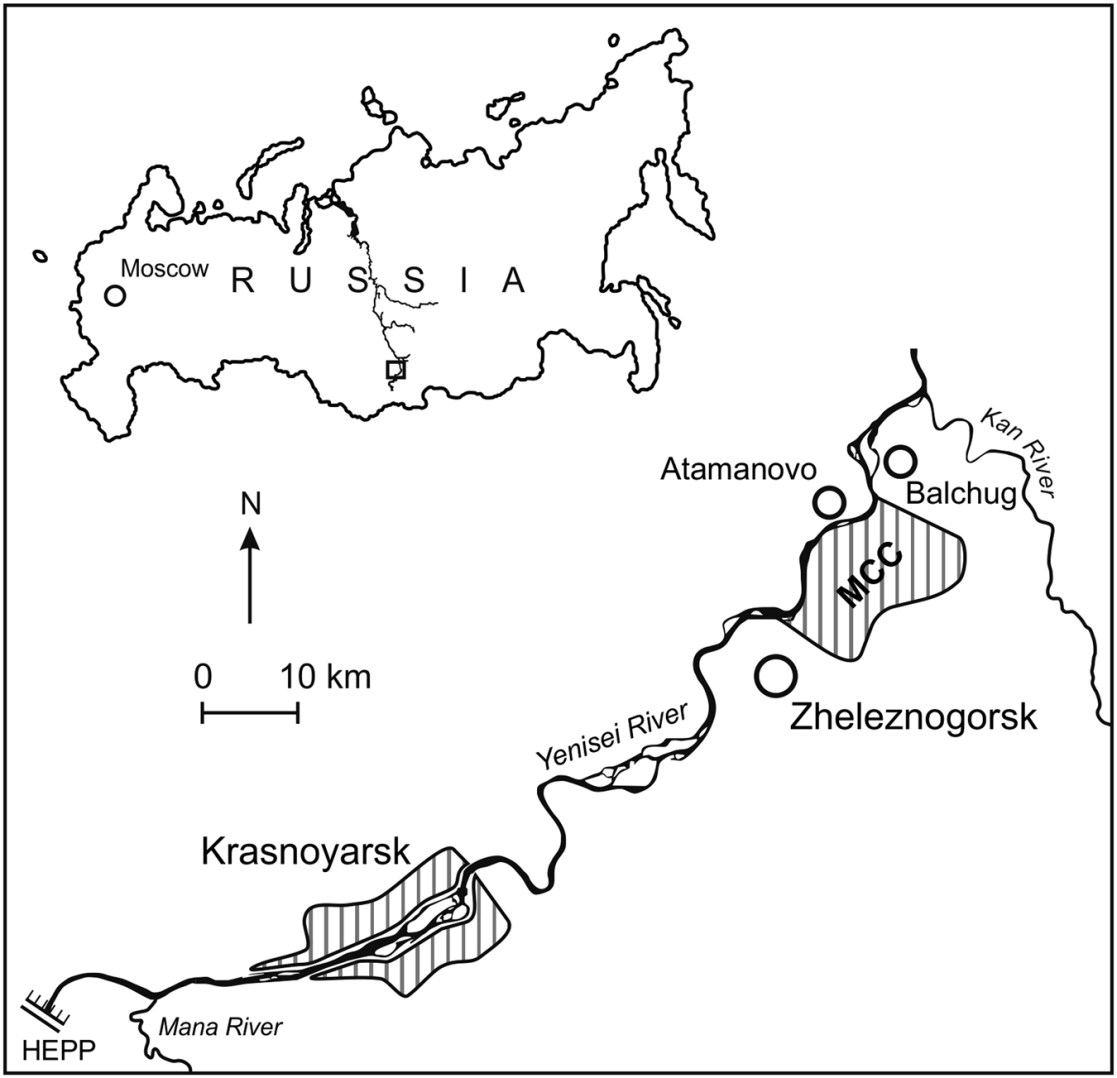
Map of the south of the Krasnoyarsk Territory (Siberia, Russia), showing settlements near which the particles were found. MCC – the territory of the Mining-and-Chemical Combine; HEPP – Krasnoyarsk Hydroelectric Power Plant. The map was copied from ref. 23.

The Yenisei is one of the largest rivers in the world, flowing into the Kara Sea. The Mining-and-Chemical Combine (MCC) of Rosatom is situated on the bank of the Yenisei River, 60 km downstream from the city of Krasnoyarsk (Fig. 1). The Combine, which previously produced weapons-grade plutonium, consists of a reactor plant and a radiochemical plant. Two reactors, which had been using the Yenisei water as coolant for the reactor core, were shut down in 1992. The third reactor, which also had used the Yenisei water as coolant for some reactor parts, was shut down in spring 2010. The Combine has been in operation for about 60 years and has contaminated the downstream Yenisei River floodplain with artificial radionuclides, including transuranium elements^1–4^. From 1995 to 2016, a series of radioactive particles were identified in the floodplain soils and sediments of the Yenisei River downstream from the MCC^5–8^. Analytical characterisation showed that those particles mainly contained fission products, suggesting that they had originated from the spent nuclear fuel of the MCC reactors^5–8^. These particles were similar to fuel particles previously found in the areas contaminated with fallout from the Chernobyl accident^9^. Although no formal record of any accidents with the MCC reactors is available, a former head of the MCC reactor plant has published a book about incidents with fuel rods (melting of fuel assemblies) that led to temporary shutdowns of the reactors^10^. In addition to fuel particles, radioactive particles containing activation products were also observed in the floodplain of the Yenisei River, but their origin remains unclear^7, 8^. Due to high activities, these radioactive particles represent point sources of short- and long-term radioecological and radioanalytical significance. In previous studies, particles were mainly investigated by gamma-spectrometry and alpha-spectrometry (after radiochemical pre-treatment)^5–7^. No other state-of-the-art methods^9^, such as non-destructive analysis, were available.

The purpose of the present study was to investigate radionuclide and elemental composition of the radioactive particles from the Yenisei River floodplain using advanced analytical techniques and to discuss possible sources of these particles.

## Results

More than 200 radioactive particles were separated from the collected soil and sediments by sample splitting and gamma measurements. The size of the largest particles reached 2 mm and the mass 1 mg^6^. With time, these large fragile particles (aggregates) disintegrated physically into smaller fragments (a few microns), and it became rather difficult to extract single small particles from the radioactive samples. Other particles were more inert and were kept in the soil and sediment samples until isolation. As noted above, most of the radioactive particles contained a series of fission products^5, 6^, but particles containing mostly activation radionuclides (^60^Co and other products) were also identified^7, 8^. Hence, all radioactive particles that were found could be divided into two main classes: fuel particles and activation products.

### Fuel particles

Based on gamma-spectrometric and radiochemical analysis of the radioactive particles (Table 1), transuranics such as plutonium and americium and fission products such as caesium and strontium isotopes were present. Some of the particles contained also ^154^Eu and ^155^Eu. It is believed that these particles originate from reactor fuel^5–8^. Table 1 lists the highest concentrations of artificial radionuclides in different radioactive particles; the activity concentrations of ^137^Cs were the highest reaching up to 30,000 kBq/particle, the concentrations of ^90^Sr and ^241^Am were higher than 1,000 Bq/particle, and the concentrations of plutonium isotopes were 100 or more Bq/particle (Table 1).

**Table 1:** The highest concentrations (measured value ± counting error) of artificial radionuclides in radioactive particles detected in the Yenisei River floodplain analysed by the Institute of Biophysics, Krasnoyarsk (IBP) and the Institute of Geology and Mineralogy, Novosibirsk (IGM).

Radionuclide	Half-life, years	Fuel particles		Activation particles		
		Data of IBP	Data of IGM	Predominance of ^60^Co		Predominance of Eu isotopes Data of IGM
				Data of IBP	Data of IGM	
^137^Cs, kBq/particle	30.1	29,200 ± 900	8,500 ± 300	—	—	0.0005 ± 0.0001
^134^Cs, kBq/particle	2.1	7.3 ± 0.3	1.8 ± 0.1	—	—	—
^90^Sr, Bq/particle	28.8	1,350 ± 30	—	—	—	—
^239,240^Pu, Bq/particle	24,110 (^239^Pu) 6,560 (^240^Pu)	3.8 ± 0.2	56 ± 8	—	—	—
^238^Pu, Bq/particle	87.7	2.0 ± 0.2	119 ± 15	—	—	—
^241^Am, Bq/particle	432.2	200 ± 12	3,480 ± 100	—	—	—
^243^Am (^239^Np), Bq/particle	7,370	1.5 ± 0.1	408 ± 25	—	—	—
^60^Co, Bq/particle	5.3	—	—	53 ± 2	46,400 ± 1,500	8 ± 1
^152^Eu, Bq/particle	13.5	—	—	—	—	315 ± 22
^154^Eu, Bq/particle	8.6	—	4,800 ± 500	—	—	52 ± 10
^155^Eu, Bq/particle	4.8	—	2,000 ± 180	—	—	5 ± 2

It is interesting that the ^238^Pu concentration in the particles was higher than the ^239,240^Pu concentration, which is an anomalous fact for production of weapons-grade plutonium. The ^238^Pu/^239,240^Pu activity ratios in fuel particles varied widely from 0.1 to 60.0. This variation could be attributed to uranium fuel that has been irradiated in a reactor for a long time. As noted in a study by Cooper *et al*.^11^, the high ^238^Pu/^239,240^Pu ratios (1.4 ± 0.3) in samples of reservoir sediments contaminated by fuel reprocessing wastes from the Mayak PA complex could indicate highly enriched ^235^U fuels. Successive thermal neutron capture by ^235^U produces ^236^U and ^237^U that decays to ^237^Np. Neptunium-237 then undergoes thermal neutron capture to produce ^238^Pu. Thus, reprocessing fuel elements to recover unfissioned ^235^U can lead to nuclear wastes enriched in both ^238^Pu and ^237^Np. If such fuel elements are destroyed and disintegrated, high ^238^Pu/^239,240^Pu ratios should be expected in associated fuel particles. A previous study also showed wide variations in the ^238^Pu/^239,240^Pu activity ratios (between 0.05 and 0.4) in bottom sediment samples collected from the Yenisei River at the MCC site^2^. In addition, a ^238^Pu/^239,240^Pu activity ratio of 0.04 was reported for the surface layers of Yenisei sediments from the Yenisei Gulf^11^, which was comparable to the lower level of the plutonium isotope ratio range observed in the present work for the Yenisei sediments collected downstream and close to the MCC site.

**Figure 2:**
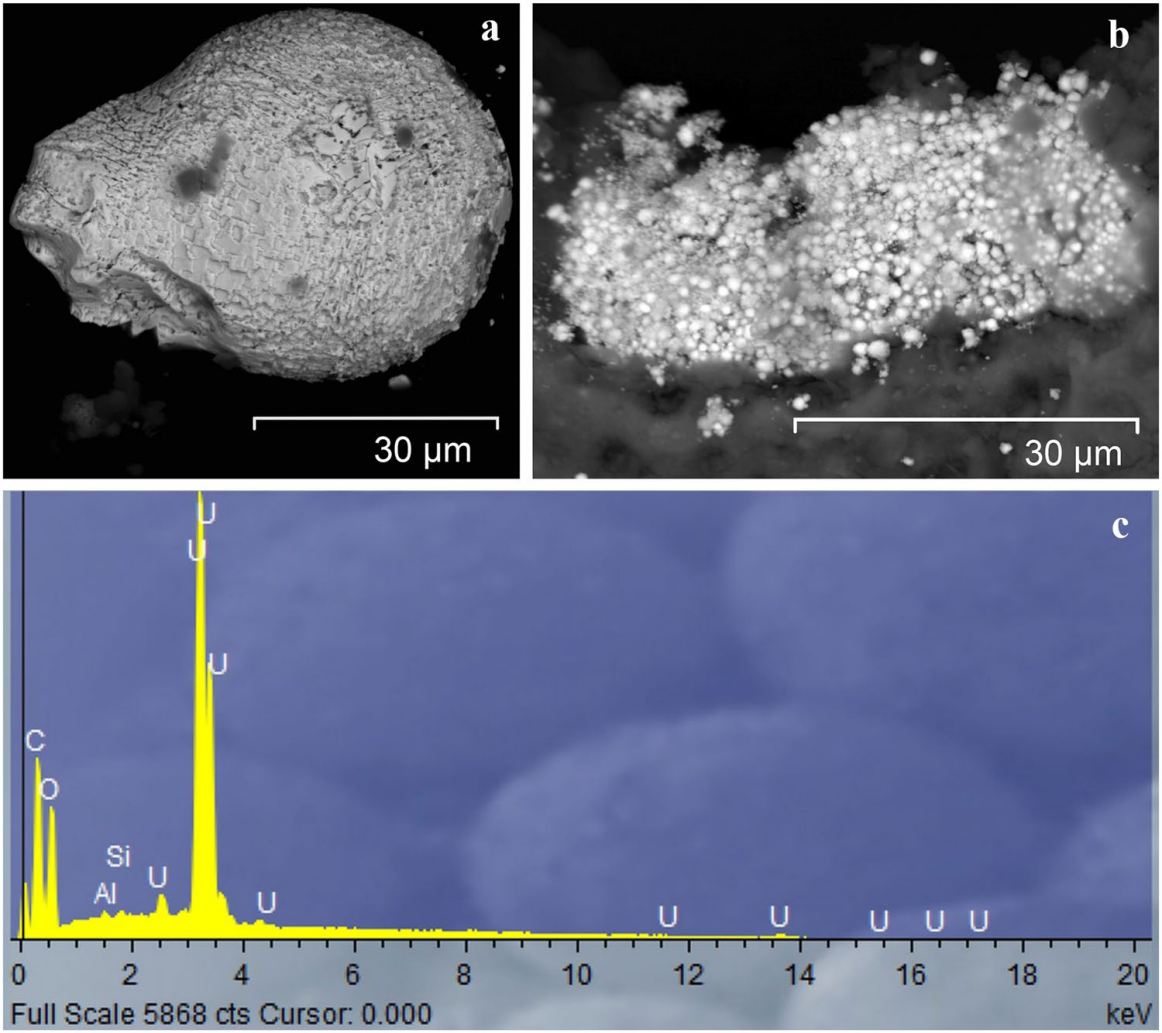
The SEM image of the typical Yenisei fuel particles investigated: (**a**) – an individual particle; (**b**) – a conglomerate of particles; (**c**) – a typical X-ray spectrum of fuel particles.

Radioactive particles with ^137^Cs activity below 1 kBq/particle were investigated using scanning electron microscopy (SEM) with X-ray microanalysis^12–14^, while the most active particles were not included in the analysis due to radiation safety. SEM images of typical Yenisei fuel particles and associated X-ray microanalysis showed the presence of uranium (Fig. 2), i.e. confirmed the fuel origin of the particles. Radioactive particles (based on the SEM image) vary in shape and size. The fuel particle in Fig. 2a is an oval-shaped uranium particle with size of approximately 50 × 60 µm. Some of the radioactive particles contained multiple inclusions in their structure – the grains of uranium compounds. Figure 2b shows the image of a part of a radioactive particle where uranium inclusions are conglomerates of numerous spherical formations of very small sizes (microns). Under certain conditions, such structures can be destroyed (by chemical dissolution or by mechanical disruption), and uranium micro-and nano-particles can migrate to surrounding soils and sediments. Then, some of the radionuclides associated with fuel particles can be released to surrounding soils (sediments).

In one of the first publications on radioactive particles from the Yenisei River^6^, the fuel particles were divided into two major groups, based on comparative analysis of ^137^Cs/^134^Cs ratios, suggesting that there had been two emergency situations at the MCC reactors, with nuclear fuel particles released into the Yenisei. The data obtained in this study indicated that the particles could be divided into three, rather than two, major groups based on comparative analysis of ^137^Cs/^134^Cs ratios. It is assumed that at the time when the particle was formed (an emergency situation), the ^137^Cs/^134^Cs ratio would be equal to 1 as observed for long term irradiated fuel. The ^137^Cs/^134^Cs ratios obtained for the Group 1 particles were between 2100,000 and 8300,000 (6 particles found in 1995 and 1998), and these particles are expected to be about 46–51 years old, i.e., they were probably formed around 1964–1969 (all data decay-corrected to 1 January 2016). For Group 2, the ^137^Cs/^134^Cs ratios ranged between 300,000 and 600,000; these particles were probably 40–43 years old, i.e., formed in 1972–1975. For Group 3, the ^137^Cs/^134^Cs ratios were between 4,000 and 25,000; these particles were probably 30–32 years old, i.e., formed in 1983–1985. The comparative analysis of ^137^Cs/^134^Cs ratios associated with the particles suggests that over 60 years of the MCC operation, there had been at least three events (e.g., emergency situations) at the MCC reactors, with release of nuclear fuel particles into the Yenisei. Emergency situations that occurred at the MCC reactors at different time points (based on ^137^Cs/^134^Cs ratios) could be the reason for the large variations in plutonium isotope ratios in fuel particles (^238^Pu/^239,240^Pu varied from 0.1 to 60.0). It is, however, difficult to find correlation between ^137^Cs/^134^Cs and ^238^Pu/^239,240^Pu activity ratios for individual particles, as we do not have the data on plutonium isotopes for all particles.

### Activation particles

In addition to fuel particles, particles containing activation products (^60^Co and other radionuclides) were also observed in the floodplain of the Yenisei River^7, 8^.

#### Activation particles containing ^60^Co

During fieldwork at the Yenisei River in 2007 and 2008, radioactive particles containing only one radionuclide, ^60^Co (cobalt particles), were found in the layers of floodplain soils close to the MCC^7, 8^. The highest ^60^Co activity concentrations in the particles reached 32,300 Bq/particle (the particle denoted by Gch-1) and 46,400 Bq/particle (the particle denoted by HP08-06) (Table 1). The cobalt particle Gch-1 was removed from the surface layer of soil mainly containing ^60^Co (18,600 Bq/kg) in August 2007. This ^60^Co concentration was almost two orders of magnitude higher than ^60^Co concentrations typical for surrounding alluvial deposits in this part of the Yenisei River floodplain (200–300 Bq/kg) and higher than concentrations of other radionuclides in this soil layer (^137^Cs - 3270 Bq/kg, ^152^Eu - 420 Bq/kg). This radioactive material may have been transported from the MCC territory during the great floods in 2006–2007. After the highly active cobalt particle (Gch-1 or HP08-06) was isolated, small fragments of the particle could still reside in the soil. During 2009–2012, microparticles that mainly contained ^60^Co of activities reaching 53 Bq/particle were detected in sediment layers both close to the MCC and at a distance of more than 800 km downstream.

**Figure 3:**
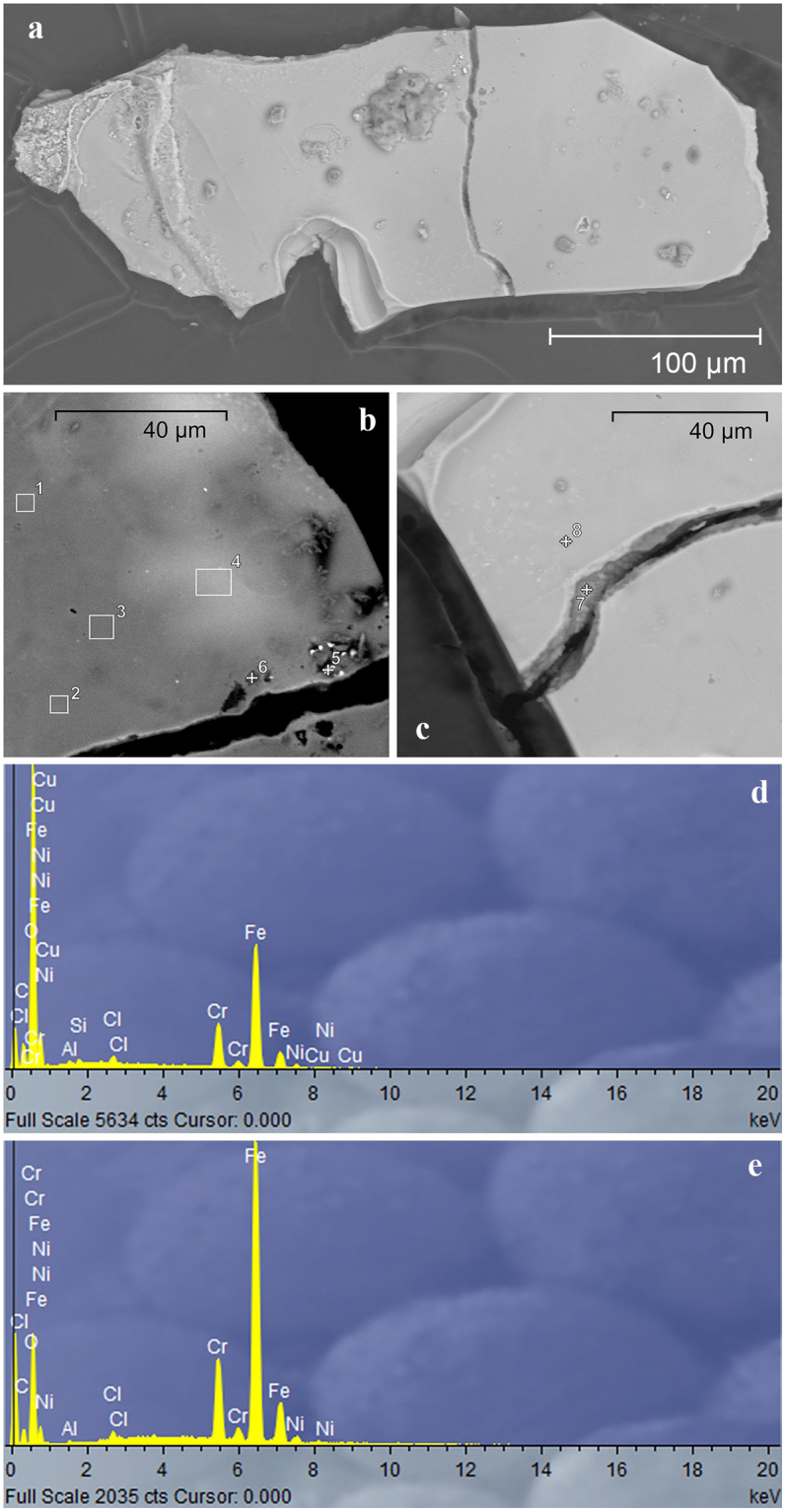
The SEM image of a typical Yenisei cobalt particle (Gch-1): (**a**) – the general appearance of the particle; (**b** and **c**) – parts of the particle at the fracture site with the points of X-ray analysis marked in the image; typical X-ray spectra of the surface (**d**) and inner (subsurface) (**e**) regions of the particle.

The probable main source of such radioactive particles is corrosion of materials in the reactor core, as the core coolant (water) carries corrosion products to the Yenisei. How long it takes for particles to form depends on the rate of corrosion of the reactor pipes and on how often the radioactive scale is removed from the reactor components. The active cobalt particles (Gch-1) were further investigated using SEM techniques (Fig. 3). The approximate size of the particle is 150 × 350 µm, and it is about 10 µm thick. The X-ray microanalysis of different regions of the particle (Fig. 3b,c,d,e; Table 2) did not reveal any substantial differences in the composition between the surface and the inner portion of the particle. The particle mainly consisted of stable elements such as Fe (up to 63%) and Cr (up to 12%); the minor components were Ni (7%), Al (2.6%), Cu (1.3%), and Ti (0.6%). The only differences were that the inner (subsurface) portion of the particle (points 5, 6 and 7) did not contain Cu, while concentrations of Ni and Al were higher than in the surface layer (Fig. 3b,c,d,e; Table 2). The main metallic materials used in nuclear power plants are stainless steels, which, in addition to Fe, contain up to 20% Cr, up to 10% Ni and some other elements (Mo, Ti, Cu etc.)^15^. Cobalt is sometimes added to alloy stainless steels, but cobalt becomes highly radioactive when exposed to the intense radiation of nuclear reactors: absorption of a neutron by stable cobalt during nuclear fission converts ^59^Co to ^60^Co. As a result, limitations are imposed on Co content in any stainless steel used in nuclear equipment. Cobalt often occurs in nature alongside nickel ore, and, therefore, nickel-containing stainless steels will contain traces of stable cobalt. The usual limit for the cobalt level in nuclear equipment is 0.2%^16^. Stainless steel pipes of MCC nuclear reactors obviously contained not only nickel (Table 2), but also cobalt, which had become radioactive over time. These data support the assumption that the source of cobalt particles most probably is scale, the corrosion product of stainless steel pipes, in the cooling system of the reactor core.

**Table 2:** Averaged normalized (to 100%) composition of the cobalt particle (Gch-1) obtained using the SEM technique at Points 1 ÷ 8 in Fig. 3b,c.

	Na	Al	Si	S	Cl	K	Ti	Cr	Fe	Ni	Cu	O	Total
1		0.43	0.42		1.00			12.55	57.95	2.19	1.29	24.17	100
2		0.50	0.45	0.30	0.95			12.10	58.09	2.19	0.95	24.47	100
3		0.31	0.49		1.07			12.33	58.47	2.22	1.00	24.11	100
4		0.55	0.41	0.32	1.04			12.21	57.41	2.59	1.01	24.46	100
5	1.60	0.44	0.45		0.35		0.58	12.19	53.04	7.03		24.32	100
6	0.61	2.61	2.78	0.28	1.14	0.75		11.06	51.85	2.11		26.81	100
7		0.28			0.53			10.25	63.26	2.03		23.65	100
8		0.33	0.38	0.28	1.05		0.43	12.38	57.75	2.18	0.92	24.3	100

#### Activation particles mainly containing europium isotopes

For the first time, particles mainly containing gamma radiation from europium isotopes (europium particles) were observed during fieldwork at the Yenisei River. Analytical characterization of these particles showed the presence of three radioactive isotopes of europium (^152^Eu, ^154^Eu, and ^155^Eu), with the activity of ^152^Eu predominating (up to 315 Bq/particle) and constituting up to 86% of the total radionuclide activity in the particles (Table 1). Some of the particles, in addition to europium isotopes, contained ^60^Co and ^137^Cs, but their activities were very low (Table 1). More than thirty europium particles were collected, in which the total radioactivity was dominated by ^152^Eu.

**Figure 4:**
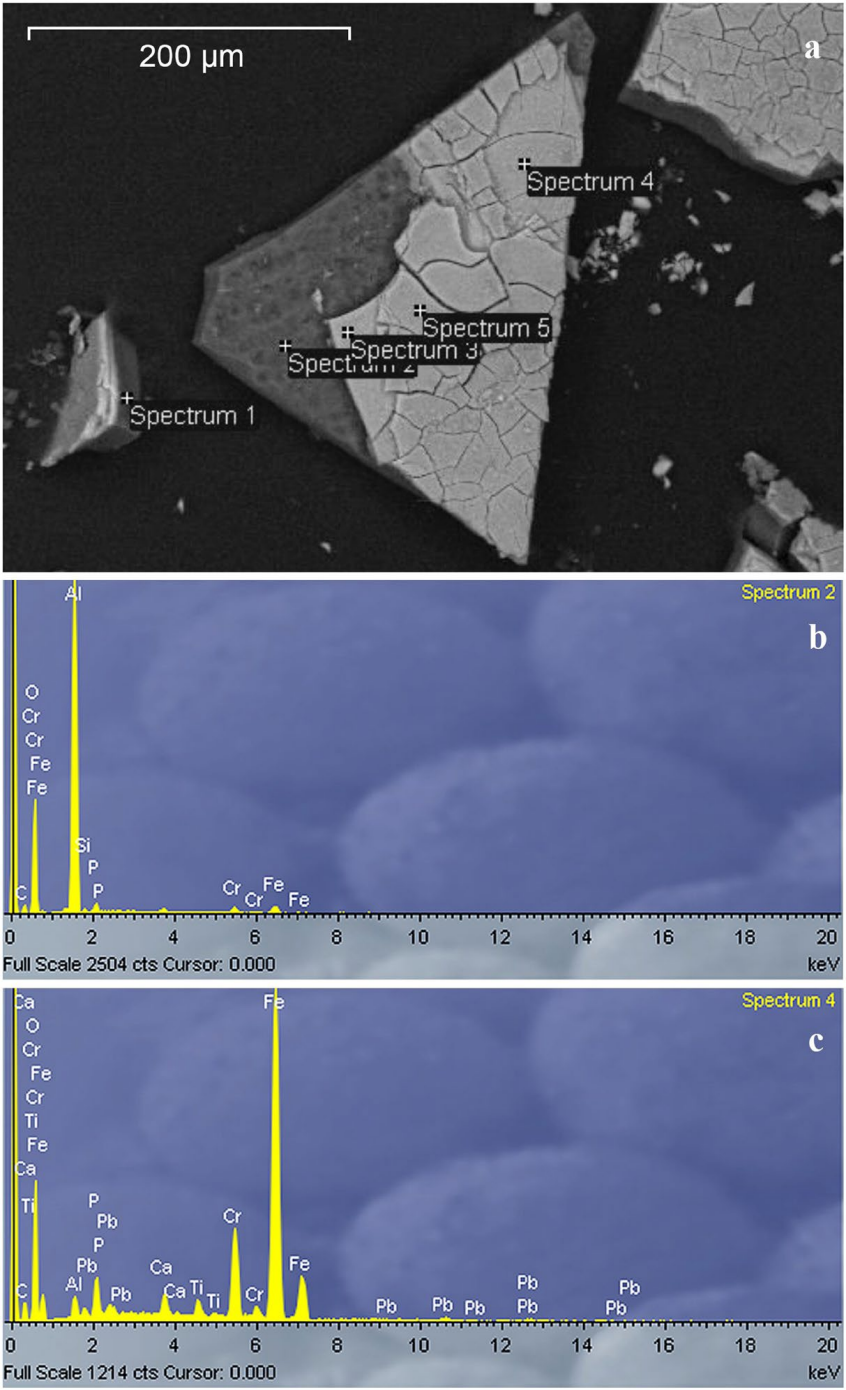
The SEM image of the typical Yenisei europium particle (Ch30): (**a**) – fragments of the particle with the points of X-ray analysis marked in the image; typical X-ray spectra of the inner (subsurface) (**b**) and surface (**c**) regions of the particle.

All europium particles have similar structure: particles are sharp and flat, without any signs of weathering or rounding (Fig. 4). Their thickness ranges between 10 and 40 µm. Geometrical dimensions reach 1750 µm. It is easy to isolate them from the surrounding soil not only because they are large, but also because of their different optical appearance compared with surroundings. Several europium particles were investigated using SEM techniques. Figure 4 shows the SEM image of one of the particles (Ch30), whose size is 200 × 400 µm and the total thickness is 30–35 µm. Particle Ch30 is a flat multilayered object. X-ray microanalysis of different regions of the particle showed (Fig. 4) considerable differences in the composition between the surface and the inner layer of the particle. The inner glass-like layer, of thickness 20–25 µm, consists mainly of aluminium, with minor iron compounds (Fig. 4a,b). The surface layer, of thickness 5–15 µm, consists mainly of compounds of iron and chromium, with minor components such as titanium, lead, and aluminium (Fig. 4a–c). The level of europium in this particle was surprisingly below detection limit of the SEM-EDX, and no traces of uranium could be observed. The particle was very fragile and easy to break. During preparation for the SEM-EDX analysis, the initial large particle was broken into many pieces with sizes between several hundred microns and a few microns. This can explain the wide occurrence of europium particles in the contaminated soils that were examined. The elemental composition of the particle matrix suggests low solubility in natural conditions.

The origin of the europium containing particles is not clear. Naturally occurring europium is known to consist of two stable isotopes – ^151^Eu and ^153^Eu, which constitute 47.77 and 52.23%, respectively. Both isotopes have relatively high neutron capture cross sections. Therefore, europium remains effective for a long time as an absorbent material of control rods in the reactor core. These control rods serve as a basis of reactor control and protection systems. As europium absorbs neutrons, it produces a chain of daughter isotopes (^152^Eu, ^154^Eu, and ^155^Eu), which have higher neutron capture cross sections than the initial isotopes, ^151^Eu and ^153^Eu. Europium-based neutron absorbing materials have been used in the USSR and Russia (although nowhere else in the world) since the late 1950s. Researchers from the Research Institute of Atomic Reactors noted^17, 18^ that europium-based compounds such as europium oxide or europium oxide embedded in metallic matrices were commonly used in control rods of nuclear reactors in Russia. For example, such control rods have been used in reactors in nuclear icebreakers that have been operated for more than 20 years. High gamma activities of ^152^Eu and ^154^Eu are assumed to have been generated in self-contained reactors over that time. In addition, europium absorbers have been used in the SM-2 and MIR research reactors and in the BN-350, BN-600, and BOR-60 fast reactors (Russia). Thus, storage and reprocessing of control rods have become an issue, as high activity concentrations of, for instance, europium isotopes (up to 2.2 10^12^ Bq/g) with relatively long half-life (up to 13 years) have been accumulating over time.

Risovany *et al*. reported^19^ that control rods based on europium oxide dispersions in metal matrices such as Al + Eu_2_O_3_ or Co + Eu_2_O_3_ compositions had been successfully used in nuclear reactors. The authors reported that radiation from the reactor and temperatures higher than 800 °C could cause thermal stresses in Eu_2_O_3_. As europium oxide products are brittle, such radiation and temperature stress can be sufficient for the europium layer to crack and disintegrate. In addition to control rods, nuclear reactors contain compensating rods with europium-based absorbent materials. These compensating rods are constantly kept in the reactor core (for up to 13 years in the SM-2 research reactor), accumulating high ^152^Eu and ^154^Eu activities^18^. The europium containing particles detected in the Yenisei River floodplain are composed of two layers: the aluminium bulk and a coating consisting of compounds of iron and chromium and, probably minor amounts of europium. Thus, based on the assumed Al+Eu_2_O_3_ composition, these particles could possibly originate from the compensating or control rods. As europium in the particles was below the SEM-EDX detection limit, it is possible that the brittle europium-containing layer could have been destroyed by the flow of neutrons and high temperature. Then, particles with minor amounts of Eu, but still identified by the gamma emitters, were probably carried by the reactor core coolant (water) to the Yenisei River.

## Discussion

The technical document of IAEA of 2011 “Radioactive Particles in the Environment: Sources, Particle Characterization and Analytical Techniques”^9^ is a review concerning all kinds of radioactive particles that have been identified so far in the environment, with focus on the sources that have contributed to the release. The main sources of radioactive particles in the environment are different parts of the weapon and nuclear fuel cycles such as nuclear weapon tests, nuclear weapon and reactor accidents, dumping of radioactive waste, and releases from reprocessing facilities. Although the information has been scarce, the document includes radioactive particles in the floodplain of the Yenisei River and their potential source – direct effluents from the Mining and Chemical Combine in Krasnoyarsk. The Rosatom officials repeatedly declared that, in contrast to other similar plants (the “Mayak” Production Association in the Chelyabinsk Oblast and the Siberian Chemical Combine in the Tomsk Oblast), the MCC had an accident-free track record. However, we have isolated more than 200 radioactive particles in the floodplain of the Yenisei River downstream from the MCC site. Based on autoradiography, more than 250 particles were identified in a 0.36 g ^137^Cs-containing soil sample. The occurrence of radioactive particles, varying in size, in the region close to the MCC is estimated by MCC officials^20^ to be very high, reaching 1 × 10^10^ particles per km^2^ in the 0.5 m soil layer. Thus, a large number of radioactive particles are most probably still present in the soils and sediments downstream from MCC in areas open to public.

The present results suggest that the nuclear fuel particles released into the Yenisei originate from at least three different events or emergency situations (at different time points) at the MCC reactors. Although information is scarce, incidents with fuel rods (melting of fuel assemblies) at the MCC reactor plant that led to temporary shutdowns of the reactors have been described^10^. It is logical to assume that post-incident activities (e.g., clean-up) involved removal of the damaged fuel assemblies from the reactor using different techniques, including destructive methods such as drilling through the fuel assemblies. The fuel particles investigated in the present work show large variations in the plutonium isotope ratios (^238^Pu/^239,240^Pu), indicating several different source terms. The high ^238^Pu/^239,240^Pu activity ratios in the particles may suggest damage to fuel assemblies with highly enriched ^235^U, while the low ^238^Pu/^239,240^Pu ratios may indicate damage to fuel assemblies with low-enriched uranium. It would indicate that not all post-incident activities had been performed properly. Thus, when the flow-through cooling system using the Yenisei water was eventually restarted, small parts of destroyed fuel assemblies were transferred out of the reactor channels and into the Yenisei.

As mentioned in the IAEA technical document^9^, activation particles that contained up to 50 Bq ^60^Co were found in the sediments in the Kara Sea and the fjords of Novaya Zemlya (where nuclear wastes had been dumped). SEM-EDX revealed the presence of Fe, Ni, Co, Cr, and Mn in addition to ^60^Co in a 10 × 20 μm particle. It is assumed that this ^60^Co-bearing particle may have originated from corrosion of the primary coolant system of a nuclear reactor (a so-called crud particle). The ^60^Co particles found in the Yenisei River had higher activity, reaching 46,400 Bq/particle, and these were not individual particles.

The europium containing particles detected in the Yenisei River seem unique, as no reports on such particles are available. As europium in the particles could not be observed by the SEM-EDX system used here, more sensitive techniques are needed to quantify the amount of Eu/particle. However, the gamma emitting Eu isotopes identified in the particles are without doubt originating from the reactor. Thus, it is assumed that Eu is probably released from damaged reactor compensating or control rods at the MCC.

The presence of radioactive particles from different sources (fuel, cobalt, and europium particles) in the Yenisei River floodplain makes this region a unique site for studying environmental effects of the particles. These radioactive particles represent point sources of short- and long-term radioecological and radioanalytical significance. Direct effects relate to internal doses following inhalation of respiratory particles or ingestion of particles via the food chain, as well as skin doses received from surface contamination. Long-term effects relate to ecosystem transfer of radionuclides remobilized from radioactive particles over time, due to weathering.

## Materials and Methods

### Particle collection

Soil and sediment samples containing particles were collected in the Yenisei floodplain during 2002–2012. The procedure of particle collection includes in-field gamma measurements to identify anomalously high radioactivity (hot spots) followed by sampling of bulk material (soil/sediments). All soil samples were dried at room temperature, and then a combination of numerous sample splittings and sequential radioactivity measurements (gamma) was used to detect and isolate particles. The procedure of splitting and counting is repeated until a sufficient small fraction containing the radioactive particle is reached. In the present work, portable devices with scintillation crystals (SRP-97 survey radiometers, Russia) were utilised for the field measurements. In the laboratory, samples of minimal mass and maximal activity (due to the presence of the particle) were isolated by sample splitting using an SRP-97 radiometer and a Radiagem 2000 portable dose rate meter (Canberra, USA). Then, the active soil (sediment) samples containing particles were placed under a binocular microscope or a light microscope, to visually isolate radioactive particles as their colour or shape differed from the surrounding soil particles. Samples were screened for heterogeneities using digital phosphor imaging (image plate and image plate scanner, Typhoon 8600, Molecular Dynamics) to detect small-size radioactive particles.

### Gamma-ray spectrometry

The activity concentrations of several radionuclides (^60^Co, ^134^Cs, ^137^Cs, ^152^Eu, ^154^Eu, ^155^Eu, ^241^Am, etc.) in the particles were measured using a Canberra γ-spectrometer (USA) coupled to a GX2320 hyper-pure germanium detector with 23% relative efficiency. A resolution (full width at half-maximum, FWHM) for the detectors was estimated at 1.97 keV for the 1332.5 keV line of ^60^Co. The counting time was chosen in order to reduce the uncertainty, due to the random process of decays, to less than 5% for all analytical γ-lines. The γ-ray spectra were analysed using the GENIE-2000 software (Canberra). In addition, γ-spectrometric analysis was also performed using an EGPC192-P21-R low background well-type HPGe detector (EURISYS MEASURES, France) with relative efficiency of 50%, FWHM (1,332 keV) – 2.1 keV. For particles with high activities of radionuclides (mainly ^137^Cs), the dead time value was kept within the working range of the spectrometric route by changing the measurement geometry (increased distance to the detector). The efficiency curve for each geometry was determined by using a standard kit of spectrometric point γ-sources (^152^Eu, ^60^Co, ^241^Am, ^137^Cs, ^134^Cs and ^133^Ba) with certified values of radionuclide activities (JSC Isotope, Moscow). The detection limits for all measured isotopes were of the order of 0.002 Bq.

### Scanning electron microscopy techniques

The surface elemental composition and micro-morphology of the particles were examined using a MIRA 3 LMU scanning electron microscope (Tescan Orsay Holding) with Aztec Energy/INCA Energy 450+ XMax 80 and INCA Wave 500 microanalysis systems (Oxford Instruments Nanoanalysis Ltd). Particles were further localised and characterised using a Jeol 840 Scanning Electron Microscope and a Zeiss EVO 50 variable pressure Environmental Scanning Electron Microscope (ESEM), both with Energy Dispersive X-ray analysis (SEM-EDX) systems attached.

### Measurements of plutonium isotopes and ^90^Sr

The activity concentration of plutonium in the particles was measured using a standard procedure^21^. The particle was dissolved in boiling 8 M HNO_3_ in the presence of the catalyst, KBrO_3_. To determine chemical yield, ^242^Pu or ^236^Pu was used as a tracer. The filtrates of the solutions were heated to 90 °C in the presence of NaNO_2_ to stabilize plutonium in the Pu^4+^ form. Then, the solution was sorbed on an anion-exchanger AV-17-8, and 100 ml 7.5 M HNO_3_, 100 ml 10 M HCl and 50 ml 7.5 M HNO_3_ were sequentially passed through columns with the sorbent. Then, plutonium was eluted with 25 ml of the 0.01 M HF solution in 0.035 M HNO_3_. The resultant solution was evaporated, and plutonium was electrolytically deposited onto the polished stainless steel target disc. After deposition, the target disc was measured using an α-spectrometer. The chemical yield was about 60%. Alpha-spectrometric analysis of plutonium isotopes was performed using alpha-spectrometers: 1) 7184 with a PLUS 300-15 high-resolution silicon detector (EURISYS MEASURES, France) and 2) an ALPHA-ENSEMBLE-8-RM all-in-one 8-chamber alpha spectrometer with ENS-U300 semiconductor ion-implanted silicon detectors (Ametek – ORTEC). The average measurement time was 24 h per sample. The detection limit for ^239,240^Pu and ^238^Pu was less than 0.001 Bq.

To determine isotopes of plutonium and strontium, selected particles were dissolved in boiling 8 M HNO_3_ + 0.2 M KBrO_3_, and then the resulting solutions were evaporated to wet salts. The plutonium chemical yield was determined by ^236^Pu tracer. Plutonium was concentrated and purified by using a chromatographic column packed with VP-1AP anion exchanger following the procedure described in detail elsewhere^6, 22^. Then, plutonium was determined using alpha-spectrometer (Canberra)^6^. To determine strontium after determination of plutonium, the solution was concentrated to wet salts and diluted with 2 M HNO_3_. Then, ^90^Sr was separated on a column with DCH18C6 solid extractant (styrene/divinylbenzene co-polymer matrix)^6^. After extraction, the column was washed with 2 M HNO_3_, and strontium was eluted with hot distilled water. Then, ^90^Sr was deposited onto a nuclear filter and its activity was measured with a low-background gas ionization detector (Tesla)^6^. The chemical yields for Pu and Sr were 50 and 65%.
